# Molecular characterization of exosome-like vesicles from breast cancer cells

**DOI:** 10.1186/1471-2407-14-44

**Published:** 2014-01-27

**Authors:** Stefan Kruger, Zakaria Y Abd Elmageed, David H Hawke, Philipp M Wörner, David A Jansen, Asim B Abdel-Mageed, Eckhard U Alt, Reza Izadpanah

**Affiliations:** 1 Applied Stem Cell Laboratory, Heart and Vascular Institute, Department of Medicine, Tulane University Health Sciences Center, 1430 Tulane Avenue, SL-48, Room 9520, New Orleans, LA 70112, USA; 2 Department of Urology, Tulane University Health Sciences Center, 1430 Tulane Avenue, SL-48, Room 9520, New Orleans, LA 70112, USA; 3 Department of Molecular Pathology, University of Texas M.D. Anderson Cancer Center, 1430 Tulane Avenue, SL-48, Room 9520, New Orleans, LA 70112, USA; 4 Department of Surgery, Tulane University Health Sciences Center, 1430 Tulane Avenue, SL-48, Room 9520, New Orleans, LA 70112, USA; 5 Isar Medical Center, Department of Medicine, Interdisciplinary Stem Cell Laboratory, Munich, Germany

**Keywords:** Breast cancer, Extracellular vesicles, Exosome, Liquid chromatography-mass spectrometry (LC-MS/MS), microRNA

## Abstract

**Background:**

Membrane vesicles released by neoplastic cells into extracellular medium contain potential of carrying arrays of oncogenic molecules including proteins and microRNAs (miRNA). Extracellular (exosome-like) vesicles play a major role in cell-to-cell communication. Thus, the characterization of proteins and miRNAs of exosome-like vesicles is imperative in clarifying intercellular signaling as well as identifying disease markers.

**Methods:**

Exosome-like vesicles were isolated using gradient centrifugation from MCF-7 and MDA-MB 231 cultures. Proteomic profiling of vesicles using liquid chromatography-mass spectrometry (LC-MS/MS) revealed different protein profiles of exosome-like vesicles derived from MCF-7 cells (MCF-Exo) than those from MDA-MB 231 cells (MDA-Exo).

**Results:**

The protein database search has identified 88 proteins in MDA-Exo and 59 proteins from MCF-Exo. Analysis showed that among all, 27 proteins were common between the two exosome-like vesicle types. Additionally, MDA-Exo contains a higher amount of matrix-metalloproteinases, which might be linked to the enhanced metastatic property of MDA-MB 231 cells. In addition, microarray analysis identified several oncogenic miRNA between the two types vesicles.

**Conclusions:**

Identification of the oncogenic factors in exosome-like vesicles is important since such vesicles could convey signals to non-malignant cells and could have an implication in tumor progression and metastasis.

## Background

Exosome-like vesicles are among small membranous extracellular vesicles (40–100 nm diameter) that are released in extracellular space [1,2]. In addition to tumor cells, the exosome-like vesicles are produced by various non malignant cell types including reticulocytes, intestinal epithelial cells, and hematopoietic cells [3]. Exosome-like vesicles are also present in body fluids such as synovial fluid, saliva, urine, semen, breast milk, and blood [4-9]. These vesicles have gained much attention for their important role in intercellular communication [3,10]. Structurally, these vesicles consist of a lipid bi-layer membrane similar to the cellular membrane, proteins including host specific proteins, mRNA, and microRNA (miRNA). Exosome-like vesicles, by transferring their content can affect various cell types [11,12]. The growing interest in the characterization of exosome-like vesicles in cancer research arises from their potential role in carrying a large array of oncogenic elements released by malignant cells such as oncogenic proteins and miRNAs. Such oncogenic proteins and miRNAs can traverse the tumor microenvironment and can be taken up by recipient non-malignant cells; this can result in the transfer of oncogenic activity [13]. For example, it has been shown that transcripts derived from glioma cells can be expressed in human brain microvascular endothelial cells upon their exosome transfer [14]. In addition to the unique signature of miRNAa in cancer cells, the oncogenic role of miRNAs has been reported in several cancers; notable examples include, the role of miRNA-155 (mir-155) in apoptosis, differentiation, angiogenesis, proliferation and epithelial-mesenchymal transfer in breast cancer [15]. Previously, it has been reported that the extracellular vesicles derived from two breast cancer cell lines, MCF-7 and 8701-BC, carry several antigens including those expressed on the cell surface such as members of integrin family, tumor associated antigens, HLA class I molecules, matrix metalloproteinase-9, and tissue inhibitors of metalloproteinase-1 [16]. In addition, the experimental evidences show that at least a number of tumor markers found in the blood circulation of breast cancer patients might be carried by extracellular vesicles [16,17]. Thus, biomarker research in breast cancer could gain great benefits from further characterization of these vesicles. In the field of breast cancer research, although the MCF-7 and MDA-MB 231 cell lines have been widely studied and characterized, there is no study analyzing miRNA and proteomics in their exosome-like vesicles.

In this study, we report the characterization of exosome-like vesicles from serum free culture medias of MCF-7 and MDA-MB 231 cell lines. The two types of exosome-like vesicles were profiled for their protein and miRNA contents. These cell lines have been shown to shed vesicles in serum-deprived media [18], thus allowing the collecting of uncontaminated vesicles in fetal bovine serum [19]. The results of this study showed a distinctive profile of the exosome-like vesicles, which could be interfering with cancer progression.

## Methods

### Cell culture and isolation of extra cellular vesicles

For the isolation of exosome-like vesicles from the two breast cancer cell lines, culture supernatants from MCF7 and MDA-MB231 cells in serum deprived DMEM media (original cell density 1×10^6^ cells/ml) were harvested. Then the exosome-like vesicles were isolated as described previously with minor modifications [20]. The culture supernatants (250 ml) were centrifuged at 300 g for 10 minutes and then at 1,200 g for 10 minutes to eliminate cells and debris. The cell-free supernatants were clarified through a 0.2 μm filter to reduce the number of contaminating large vesicles shed from the plasma membrane. The supernatants were ultra-centrifuged at 100,000 g for 60 minutes at 4°C (SW41Ti, Beckman Instruments, Fullerton CA). The pellets were resuspended in 3.6 ml PBS. Then, the vesicles were further purified using gradient centrifugation on 30% sucrose/D2O density cushion in 100,000 g ultracentrifugation (4°C for 60 minutes). A 700-μl volume of the cushion layer was collected and pelleted at 100,000 × g for 60 minutes. The pellets were washed twice with PBS, resuspended in 250 μl PBS, and stored at -80°C. Vesicular protein was measured by the Bradford assay with the Bio-Rad Protein Assay Reagent (BioRad Laboratories).

### Electron microscopy (EM)

EM imaging of vesicle preparations was performed as previously described [10], with some modifications. Briefly, vesicles (about 2 μg protein) were fixed in 1% glutaraldehyde and then layered and dried on formvar-coated 200 mesh copper grids (Polysciences, Inc. PA, USA). Grids were then stained 1% uranylacetate in water. Imaging took place at an accelerated voltage of 200 kV using a Tecnai G2 F30 TWIN, which is a 300 kV/FEG Transmission Electron Microscope.

### Protein analysis using LC-MS/MS

The exosome-like vesicles (n = 3/exosome type) were re-suspended in 100 μl of PBS, 2 μl triton X-100, and 5 μl phenylmethylsulfonyl fluoride with vortexing to dissolve the vesicles. The insoluble fraction was pelleted by centrifugation 20,000 g. The insoluble fraction was acetone precipitated at -20°C and digested in-gel with 200 ng modified trypsin (sequencing grade, Promega) for 18 hours at 37°C. Resulting peptides were analyzed by LC-MS/MS on an Orbitrap-XL mass spectrometer (Thermo Scientific, Waltham MA). Proteins were identified by database searching of the fragment spectra against the SwissProt (EBI) protein database using Mascot (v 2.3, Matrix Science, London, UK). Typical search settings were: mass tolerances, 10 ppm precursor, 0.8d fragments; variable modifications, and methionine sulfoxide, pyro-glutamate formation; up to 2 missed cleavages. The MS/MS spectra were then searched against the NCBI human reference sequence database with the search program MASCOT, a mass spectral search algorithm that uses mass spectrometry data to identify proteins from primary sequence databases. (Matrix Science, Boston, MA). The identified peptide features were matched to a reference database and were scored according to the probability of an overlap between the peptide feature and the database peptides resulting in a ranked list of possible peptide. This analysis generated ion scores [ions score = 10*Log (P), where P is the probability that the observed match is a random event] for each peptide feature. Individual ions scores > 38 indicate identity or extensive homology (p < 0.05) were considered.

### Western blot analysis

Exosome-like vesicles were lysed in 40 μL of lysis buffer (Promega) containing 1 μL of proteinase inhibitor cocktail (Sigma). The total protein concentration was measured using a Bradford assay containing Coomassie Plus protein reagent (Bio-Rad Laboratories) according to the manufacturer’s specifications. Equivalent amounts of total lysate were subjected to SDS-PAGE using 10% polyacrylamide gels. Proteins were electroblotted to polyvinylidene difluoride membrane (Millipore). The membranes were then blocked and incubated in anti-Annexin A2 (rabbit polyclonal; Abcam), Alpha-enolase (mouse monoclonal; Santa Cruz), Anexin A1 (mouse monoclonal; Abcam), and EpCAM (mouse monoclonal; Abcam). Alkaline phosphatase–conjugated anti-mouse or anti-rabbit IgGs were used as secondary antibodies (Bio-Rad) for detection. Then the membranes were incubated with Western Blotting Detection Reagents (Bio-Rad) according to the manufacturer’s instructions and exposed to autoradiography film.

### miRNA isolation, profiling, and microarray data analysis

RNA was isolated from exosome-like vesicles using the mirVana miRNA Isolation Kit (Ambion). Then the RNA samples were quality-checked via the Agilent 2100 Bioanalyzer platform (Agilent Technologies). The results of the Bioanalyzer run were visualized in a gel image and using the Agilent 2100 Bioanalyzer expert software, the RNA Integrity Number (RIN) was evaluated. This checks the integrity and overall quality of total RNA samples. The samples with RIN number of >6 were selected for miRNA microarray experiments [21]. The microarray data analysis was performed as published previously [22]. Briefly, normalization and calculations of sample versus Universal Reference ratios were performed with miRXploreR software (Miltenyi Biotec) according to the calibration oligonucleotide method (the median Cy5/Cy3 signal intensity ratio of the spiked in miRcontrol calibration-oligos served as reference values). Subsequently, re-ratios between MDA-Exo samples relative to the in silico pooled reference MCF7-Exo were generated by multiplying each signal ratio of sample MDA-Exo with the reciprocal value of the miRNA signal ratio of the pooled MCF7-Exo samples. A p-value indicating the reliability of the re-ratio value was calculated for each miRNA based on the individual signal intensities relative to background for the co-hybridized samples.

Candidate miRNAs with differential expressions between the MDA-Exo samples and the MCF7-Exo samples were selected by a re-ratio p-value ≤0.0001 and at least two-fold change in at least one comparison (Accession #GSE52802). Records, which may correspond to questionable miRNAs according to Chiang et al., [23] or in-house validated miRNAs were removed from the candidate list. The resulting expression profiles of the selected miRNAs were hierarchically clustered using TIGR MeV [24]. One-dimensional hierarchical clustering [25] was applied using Euclidean Distance and complete linkage method. Also the miRNAs were quantified using the Universal Reference (UR) consisting of 954 synthetic miRNAs in equimolar concentrations, which enables the cross-referencing of experiments. To discriminate questionable results from relevant results, the absolute quantification was performed only for those miRNAs which revealed a signal in the UR sample as well as in the sample of interest at least 1-fold above average signal intensities of the background [23].

For the validation of the miRNA array we performed RT-PCR to assess the miRNA levels of selected miRNA from the let-7 family using SYBRgreen MasterMix (Exiqon, Vedbaek, Denmark). Primers were used for Let-7a: ugagguaguagguuguauaguu; Let-7b: ugagguaguagguugugugguu; Let-7c: ugagguaguagguuguaugguu; Let-7d: agagguaguagguugcauagu; Let-7e: ugagguaggagguuguauagu; Let-7f: ugagguaguagauuguauaguu; Let-7i: ugagguaguaguuugugcugu. Relative expression levels were calculated based on the expression of three constitutive (u6, 5 s, snord44) miRNA references. Expression levels of miRNAs were calculated after subtracting the CT-values of the endogenous references, and fold change of gene expression was subsequently calculated using ΔΔCT-method.

### Statistical methodology

All data were summarized using descriptive statistics such as mean and standard deviation. The variance method was used to compare the mean differences. Where meaningful, the results were presented graphically. The study hypotheses were tested at 5% level of significance throughout the analysis. Estimates of means and their 95% confidence intervals were calculated. R-computing software was used to plot the graphs.

## Results

### Isolation of exosome-like vesicles from MCF7 and MDA-MB 231 cells

**Figure 1:**
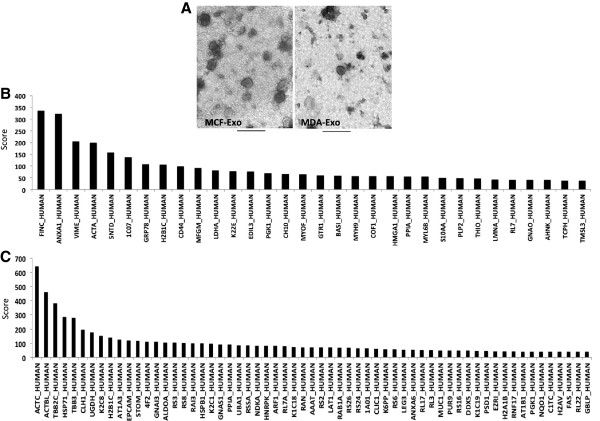
**Characterization of morphology and protein content of exosome-like vesicles. A)** MCF-Exo and MDA-Exo were negatively stained using uranyl acetate and viewed by electron microscopy. Inside images are with higher magnification, the scale bar represents 500 nm. **B)** Identified peptide targets exclusively expressed in MCF-Exo or **(C)** MDA-Exo. The peptide targets were identified using Mascot protein database. The score is the probability of an overlap between the peptide feature and Mascot database (p < 0.05).

The exosome-like vesicles were collected and purified from the culture supernatants of two breast cancer cell lines, MDA-MB 231 and MCF-7. The protein assessment of the exosome-like preparations indicated of quiet similar amounts of vesicles for both MCF7 (25.594 ± 2.16 μg/ml) and MDA-MB 231 cells (24.388 ± 3.72 μg/ml). Electron micrographs revealed that the isolated exosome-like particles consisted of primarily round shaped vesicles (Figure 1A). Quantitative analysis indicates that MCF-7 derived exosome-like vesicles (MCF-Exo) and MDA-MB231 derived exosome-like vesicles (MDA-Exo) exhibit relatively similar size with the diameter ranging from 80 to 200 nm.

### Proteomic analysis of exosome-like vesicles derived from MCF-7 and MDA-MB 231 cells

**Figure 2:**
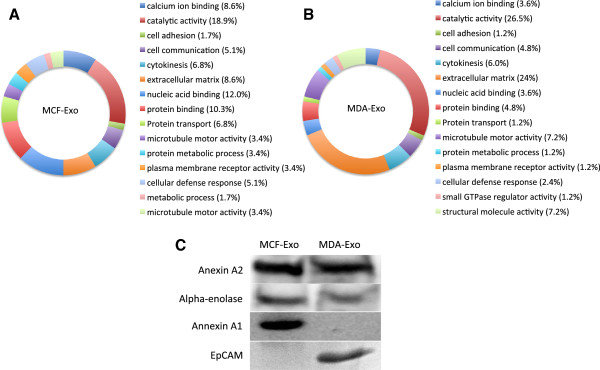
**Exosome-like vesicle proteomics analysis.** Diagram illustrating the distribution of proteins identified from MCF-Exo and MDA-Exo. Gene ontology analysis identified proteins in MCF-Exo **(A)** and MDA-Exo **(B)** using PANTHER software. The proteins detected in MCF-Exo and MDA-Exo were grouped according to their putative functions and are shown by percentage of total identified proteins. **C)** Western blot analysis of Annexin A1, Annexin A2, alpha-enolase, and EpCAM expression in exosome-like vesicles.

To profile the protein content of the two types of exosome-like vesicles, the vesicles were treated with acetone to precipitate their protein content. Then, 15 μg of total protein was loaded on SDS-PAGE gel, which was followed by in-gel trypsin digestion, and was subsequently analyzed by LC-MS/MS. The data analysis using Mascot database has identified proteins and their scores for both MCF-Exo and MDA-Exo. This analysis identified 59 proteins in MCF-Exo and 88 proteins in MDA-Exo. A total of 32 and 61 proteins were exclusively detected in MCF-Exo and MDA-Exo, respectively (Figure 1B and C and Additional file 1: Tables 1 and Additional file 2: Table S2). Among the identified proteins, 27 proteins found to be expressed in both types of vesicles (Table 1). The ontology of the identified proteins was analyzed using PANTHER software (Protein Analysis Through Evolutionary Relationships). As shown in Figure 2A and B, the determined proteins were grouped and compared between the two types of vesicles. All proteins were grouped into 15 protein classes including catalytic activity, cell adhesion, protein transport, and extracellular matrix. Comparing the two types of vesicles identified significant differences in the expression of extracellular matrix proteins in MCF-Exo (8.6%) and MDA-Exo (24%). This may explain the higher metastatic attribute of MDA-MB 231 compared to MCF-7 cells. While MCF-Exo contains higher nucleic acid binding and protein binding and protein transport activity, the MDA-Exo contains proteins with more catalytic activity. To validate the proteomic data, western blot analysis was performed for proteins commonly expressed in both types of exosome-like vesicles (Annexing A2 and alpha-enolase), and was also performed for exclusive expressions of Annexin A1 and EpCAM for MCF-Exo and MDA-Exo, respectively (Figure 2C).

**Table 1:** Catalogue of the identified peptide targets commonly expressed in both MCF-Exo and MDA-Exo

**Protein name**	**MW (KDa)**	**Gene**	**Average score**	**Sequence**
Actin, cytoplasmic 1	41.7	ACTB	938.5	R.GYSFTTTAER.E
				R.DLTDYLMK.I
				R.DLTDYLMK.I
				R.GYSFTTTAER.E
				R.GYSFTTTAER.E
				K.EITALAPSTMK.I
				K.EITALAPSTMK.I
Annexin A2	38.6	ANXA2	563	K.AYTNFDAER.D
				R.DALNIETAIK.T
				K.TPAQYDASELK.A
				K.TPAQYDASELK.A
				K.DIISDTSGDFR.K
				R.TNQELQEINR.V
				R.TNQELQEINR.V
				R.TNQELQEINR.V
				R.TNQELQEINR.V
Pyruvate kinase isozymes M1/M2	57.9	KPYM	340	K.GDYPLEAVR.M
				K.DITSDTSGDFR.N
				K.TPAQFDADELR.A.K
Tubulin beta chain	49.6	TBB5	258.5	K.LAVNMVPFPR.L
				K.EVDEQMLNVQNK.N
Heat shock cognate 71 kDa protein	70.8	HSP7C	252.5	K.DAGTIAGLNVLR.I
				K.NQVAMNPTNTVFDAK.R
Glyceraldehyde-3-phosphate dehydrogenase	36	G3P	249.5	R.VVDLMAHMASK.E
				K.LISWYDNEFGYSNR.V
Tubulin alpha-1C chain	49.8	TBA1C	223.5	K.DVNAAIATIK.T
				K.TIGGGDDSFNTFFSETGAGK.H
Sodium/potassium-transporting ATPase subunit alpha-1	11.2	AT1A1	197	K.TSATWLALSR.I
				R.LNIPVSQVNPR.D
14-3-3 protein zeta/delta	27.7	1433Z	194.5	R.YLAEVAAGDDKK.G
				R.YLAEVAAGDDKK.G
				K.SVTEQGAELSNEER.N
				K.GIVDQSQQAYQEAFEISK.K
Histone H4	11.3	H4	181.5	R.ISGLIYEETR.G
				K.TVTAMDVVYALK.R
				R.DNIQGITKPAIR.R
				R.DNIQGITKPAIR.R
				K.TVTAMDVVYALK.R
				R.KTVTAMDVVYALK.R
Heat shock protein HSP 90-alpha	84.6	HS90A	167.5	K.DQVANSAFVER.L
				R.ELISNSSDALDK.I
				K.EDQTEYLEER.R
				R.GVVDSEDLPLNISR.E
				R.NPDDITNEEYGEFYK.S
Heat shock protein HSP 90-beta	83.2	HS90B	156	K.EQVANSAFVER.V
				K.EDQTEYLEER.R
				R.GVVDSEDLPLNISR.E
				R.NPDDITQEEYGEFYK.S
Brain acid soluble protein 1	22.6	BASP1	155.5	K.AEPPKAPEQEQAAPGPAAGGEAPK.A
				K.AEPPKAPEQEQAAPGPAAGGEAPK.A
				K.AAEAAAAPAESAAPAAGEEPSKEEGEPK.K
				K.AQGPAASAEEPKPVEAPAANSDQTVTVKE
Elongation factor 1-alpha 1	50.1	EF1A1	128.5	K.IGGIGTVPVGR.V
				K.STTTGHLIYK.C
				K.STTTGHLIYK.C
				K.STTTGHLIYK.C
				K.STTTGHLIYK.C
Myristoylated alanine-rich C-kinase substrate	31.5	MARCS	118	K.EAPAEGEAAEPGSPTAAEGEAASAASSTSSPK.A
				K.EELQANGSAPAADKEEPAAAGSGAASPSAAEK.G
Annexin A5	35.9	ANXA5	98	K.VLTEIIASR.T
				R.SEIDLFNIR.K
Calmodulin	16.8	CALM	96.5	K.EAFSLFDKDGDGTITTK.E
				R.VFDKDGNGYISAAELR.H
MARCKS-related protein	19.5	MRP	90	K.GEGESPPVNGTDEAAGATGDAIEPAPPSQGAEAK.G
				R.GDVTAEEAAGASPAK.A
Integrin alpha-2	129.2	ITA2	76	K.TQVGLIQYANNPR.V
Galectin-3-binding protein	65.3	LG3BP	73	R.ASHEEVEGLVEK.I
Histone H1t	22	H1T	60.5	K.ALAAAGYDVEK.N
Alpha-enolase	47.1	ENOA	60.5	R.YISPDQLADLYK.S
Kinesin-like protein KIF12	70.6	KIF12	60	K.LTKLLADSLGGR.G
Elongation factor 1-gamma	50.1	EF1G	57	K.ALIAAQYSGAQVR.V
Peroxiredoxin-1	22.1	PRDX1	50.5	K.ATAVMPDGQFK.D
				R.QITVNDLPVGR.S
Ubiquitin-40S ribosomal protein S27a	17.9	RS27A	49.5	K.ESTLHLVLR.L
14-3-3 protein epsilon	29.1	1433E	43.5	K.EAAENSLVAYK.A

The peptide targets were identified using Mascot database. The score is the probability of an overlap between the peptide features and the database peptides (p < 0.05).

### microRNA analysis

**Figure 3:**
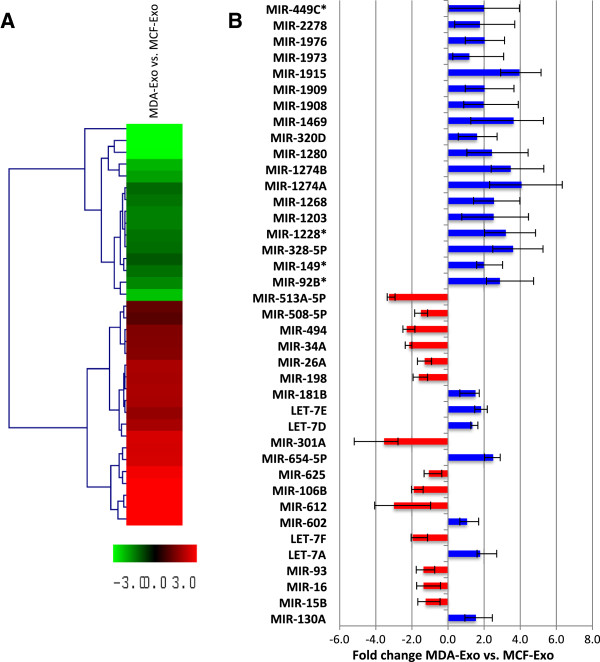
**Differential gene expression in exosome-like vesicles. A)** Hierarchical clustering was used to display miRNAs differentially expressed in each vesicle type. The extent of green (decreased fold change) or red (increased fold change) colors is directly proportional to the magnitude of differential expression of miRNAs. To perform these comparisons, probe sets whose target was not detected in any sample were eliminated from the data matrix. The data were grouped by type of exosome-like vesicles and members of each group were pooled, before a Student’s *t*-test was used to identify those miRNAs that were expressed in a statistically significant manner (P < 0.05). **B)** miRNA profiles of the MDA-Exo versus MCF-Exo. Bars represent fold change of hybridization signals in MDA-Exo against MCF-Exo. Blue and red bars display relatively higher miRNA expressions in MDA-Exo and MCF-Exo, respectively. The miRNAs were quantified using the universal reference in equimolar concentrations and cross referenced with experimental data. Then the expression values were compared in two types of exosome-like vesicles (three independent experiments; P < 0.05).

Global miRNA comparison of two types of exosome-like vesicles was performed to obtain an overview of differences in miRNA expression patterns that may play a role in the manifestation of the two breast cancer cell types. These experiments were performed using miRNA microarray (Miltenyi Biotec), and the data were analyzed by normalization and calculations of sample intensity versus the Universal Reference, using miRXploreR software (Miltenyi Biotec), according to the previously described method [22]. Hierarchical clustering was used to display miRNAs that are differentially expressed in each type of exosome-like vesicles (Figure 3A). The quantification on miRNAs using cross-referencing of miRNA signals against universal reference revealed higher amounts of mir-198, mir-26a, mir-34a and mir-49a enclosed in MCF-Exo, whereas, several miRNAs including mir-130a, mir-328, and mir-149 were more abundant in MDA-Exo (Figure 3B).

**Figure 4:**
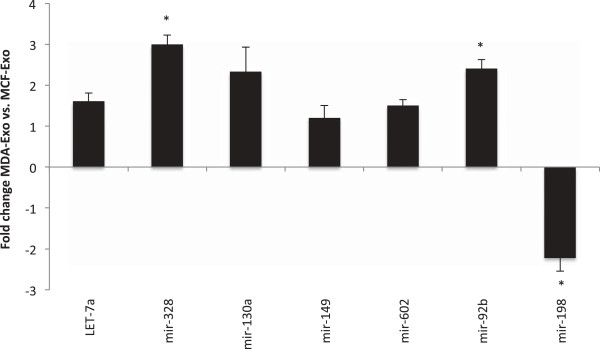
**Quantitative real-time PCR of miRNAs performed to validate the microarray data.** Down-regulation of mir-198 in MCF-Exo and up-regulation of let-7a, mir-328, mir-130a, mir-149, mir-603, and mir-92b from microarray experiments were validated by quantitative PCR analysis (*P < 0.01). miRNA expression values were normalized to three constitutive miRNA references u6, 5 s, snord44 (five independent experiments; P < 0.05).

Then to validate the microarray data, a group of miRNAs (let-7a, mir-328, mir130a, mir-149, mir-602, mir-92b, and mir-198) was selected for RT-PCR analysis. The miRNAs were readily detected with relatively high amounts in both types of vesicles. Comparing the two types of exosome-like vesicles showed that while MCF-Exo encloses greater amounts of mir-198, MDA-Exo contains higher levels of let-7a, mir-328, mir-130a, mir-149, mir-602, and mir-92b (Figure 4).

## Discussion and conclusions

This study reports the characterization of exosome-like vesicles released from two breast cancer cell lines, MCF-7 and MDA-MB 231. The results of this study are important when considering circulating exosome-like vesicles for diagnosis, and assessing the biological significance of the release of onco-proteins and miRNAs from these vesicles. This becomes more significant in view of the fact that exosome-like vesicles can easily enter the circulation and may possibly affect non-malignant cells. The MCF7 and MDA-MB 231 cell lines account for a large number of basic and pre-clinical studies on breast cancer around the globe [26]. Several studies indicated a possible role of extracellular vesicles in tumor progression; however, the exact protein and genetic attributes enclosed in these vesicles remain to be determined. The circulating vesicles have been described in patients with various tumors [8,27,28], suggesting that they may serve as a diagnostic and prognostic tool. In the context of cancer, the potential role of tumor-derived extracellular vesicles in tumor microenvironment and their involvement in cancer progression must be considered. Detailed interrogation of the protein dataset revealed a protein signature of exosome-like vesicles, which may further delineate their biogenesis. A number of studies have detailed the proteomics [29] and miRNA profile of MCF-7 [30] and MDA-MB 231 cells [17]. However, the present study is the first report comparing these two cell lines for their both proteomic and miRNA profiles. Proteomic analysis identifies several proteins expressed in both MCF-Exo and MDA-Exo, including proteins belonging to the Annexin family. Annexins are calcium-dependent phospholipid-binding proteins that play an important role in the regulation of cellular growth and in signal transduction pathways. Histone H4 protein is expressed in both types of exosomes, which has a crucial role in epigenomic alterations of cells via disturbing normal expression of DNA methyltransferase and histone methyltransferase. This is associated with increased malignant properties of cancer cells [31]. Calmodulin, a regulator of Akt pathway is associated with poor prognosis in breast cancer patients [32], has been identified in both studied types of exosome-like vesicles. Comparing the MCF-Exo and MDA-Exo demonstrates a significantly higher expression of matrix metalloproteinase proteins in MDA-Exo. This can be related to the enhanced metastatic characteristics of MDA-MB 231 cells. In contrast, the MCF-Exo contains higher levels of nucleic acid, protein binding, and transfer proteins. In addition, the significant Gene Ontology analysis revealed that several of profiled miRNAs are related with pathways which may play an important role in tumor formation. For example, comparing the miRNAs in MDA-Exo to MCF-Exo showed a higher expression of tumorigenic mir-130a in MDA-Exo. it has been shown that mir-130a contribute to tumorigenesis of colon cancer by regulating TGB-β/Smad signaling [33]. MDA-Exo also contains a significant amount of mir-328, which has been shown to target CD44, reduce cell adhesion, enhances cell migration, and regulate formation of capillary structure [34]. In contrast, MCF-Exo contains higher amounts of mir-301a. The mir-301a over expression has been implicated as a negative prognostic indicator in lymph node negative (LNN) invasive ductal breast cancer [35]. MCF-Exo also contains mir-34a, which regulates several genes including p53 [36]. The mir-106b is found in higher levels in MCF-Eox as well. This miRNA can promote breast cancer invasion and metastasis by targeting BRMS1 and RB. The mir-106b mediates TGF-β-induced epithelial-mesenchymal transfer, which is an early process of tumor metastasis [37].

For the discovery of novel circulatory tumor markers, proteomics and genomic approaches have been conducted on blood and tissue samples. However, there are contradictory reports whether profiles of miRNAa and tumor specific proteins in blood circulation are parallel with tumor’s profiles. The exosomal miRNA signatures originating from tumor cells have been reported in breast cancer or lung adenocarcinoma cases [8,38]. It is reasonable to speculate that these vesicles exert different effects to the possible acceptor targets. For example, the vesicles potentiate the malignant properties of neighboring neoplastic cells or activate non-malignant cells. Understanding the communication between the tumor cells and the extracellular environment through extracellular vesicles is of great importance. Our data show that extracellular vesicles carry oncogenic proteins and miRNAs, which may further be applicable for early detection of breast malignancy as well as delineating the possible role of extracellular vesicles in tumorigenesis and metastasis.

## Abbreviations

miRNAs: microRNAs; LC-MS/MS: Liquid chromatography-mass spectrometry; MCF-Exo: Extracellular vesicles derived from MCF-7 cells; MDA-Exo: Extracellular vesicles derived from MDA-MB 231 cells.

## Competing interests

The authors declare that they have no competing interests.

## Authors' contributions

SK, PW, and ZAE were responsible for implementing cell culture and exosome preparations. SK and DH were responsible for LC-MS/MS and protein analysis. DJ, AA, and EA were responsible for data analyses. RI and EA were responsible for experimental design. RI was responsible for the overall experimental design, data analysis, and implementation of the project. All authors read and approved the final manuscript.

## Pre-publication history

The pre-publication history for this paper can be accessed here:

http://www.biomedcentral.com/1471-2407/14/44/prepub

## Supplementary Material

Additional file 1: Table S1 List of proteins exclusively identified in MCF-Exo. Click here for file

Additional file 2: Table S2 List of proteins exclusively identified in MDA-Exo. Click here for file
