# Supplementary material for: Molecular characterization of exosome-like vesicles from breast cancer cells
Source: BMC Cancer. 2014 Jan 27;14:44. doi: 10.1186/1471-2407-14-44 (PMC3936808; doi:10.1186/1471-2407-14-44)
Supplement: Additional file 1: Table S1 — List of proteins exclusively identified in MCF-Exo. [file 1471-2407-14-44-S1.pdf]

Supplemental Table 1: List of proteins exclusively identified in MCF-Exo.

FINC_HUMAN Mass: 262622 Score: 335 Matches: 9(8) Sequences: 9(8) emPAI: 0.13
Fibronectin OS=Homo sapiens GN=FN1 PE=1 SV=4

Query	Score	Peptide
628	37	R.FTNIGPDTMR.V
803	79	R.DLQFVEVTDVK.V
883	76	K.IYLYTLNDNAR.S
884	57	K.IAWESPQGQVSR.Y
898	86	K.GLAFTDVDVDSIK.I
929	96	R.VPGTSTSATLTGLTR.G
1007	71	R.SYTITGLQPGTDYK.I
1039	68	R.GDSPASSKPISINYR.T
1070	57	R.VDVIPVNLPGEHGQR.L

ANXA1_HUMAN Mass: 38714 Score: 322 Matches: 7(7) Sequences: 7(7) emPAI: 1.08
Annexin A1 OS=Homo sapiens GN=ANXA1 PE=1 SV=2

Query	Score	Peptide
554	55	R.SEIDMNDIK.A
709	75	K.DITSDTSGDFR.N
777	81	K.TPAQFDADELR.A
1028	82	K.GTDVNVFNTILTTR.S
1166	131	R.SEDFGVNEDLADSDAR.A
1330	50	K.AAYLQETGKPLDETLKK.A
1540	66	K.GGPGSAVSPYPTFNPSSDVAALHK.A

VIME_HUMAN Mass: 53651 Score: 205 Matches: 7(6) Sequences: 7(6) emPAI: 0.58
Vimentin OS=Homo sapiens GN=VIM PE=1 SV=4

Query	Score	Peptide
561	37	R.DNLAEDIMR.L
574	73	K.FADLSEAANR.N
588	50	K.VELQELNDR.F
598	52	R.EYQDLLNVK.M
844	65	R.EEAENTLQSFR.Q
1036	63	R.TNEKVELQELNDR.F
1159	93	R.LQDEIQNMKEEMAR.H

ACTA_HUMAN Mass: 42009 Score: 200 Matches: 11(10) Sequences: 5(4) emPAI: 0.97
Actin, aortic smooth muscle OS=Homo sapiens GN=ACTA2 PE=1 SV=1

Query	Score	Peptide
488	55	R.DLTDYLMK.I
508	-46	R.DLTDYLMK.I + Oxidation (M)
647	-48	K.EITALAPSTMK.I
648	67	K.EITALAPSTMK.I
667	-39	K.EITALAPSTMK.I + Oxidation (M)
668	-53	K.EITALAPSTMK.I + Oxidation (M)
670	-49	K.EITALAPSTMK.I + Oxidation (M)
688	37	R.AVFPSIVGRPR.H
1218	-58	K.SYELPDGQVITIGNER.F
1219	61	K.SYELPDGQVITIGNER.F
1380	60	K.YPIEHGIITNWDDMEK.I

5NTD_HUMAN Mass: 63367 Score: 158 Matches: 5(4) Sequences: 4(3) emPAI: 0.38
5'-nucleotidase OS=Homo sapiens GN=NT5E PE=1 SV=1

Query	Score	Peptide
781	69	K.YPFIVTSDDGR.K
825	36	R.KVPVVQAYAFGK.Y
1152	40	K.VLPVGDEVVGIVGYTSK.E
1308	103	R.HDSGDQDINVVSTYISK.M
1313	-47	R.HDSGDQDINVVSTYISK.M

1C07_HUMAN Mass: 40648 Score: 137 Matches: 4(3) Sequences: 4(3) emPAI: 0.49
HLA class I histocompatibility antigen, Cw-7 alpha chain OS=Homo sapiens GN=HLA-C PE=1 SV=3

Query	Score	Peptide
879	38	R.YLENGKETLQR.A
921	60	K.WAAVVVPSGQEQR.Y
977	66	R.SWTAADTAAQITQR.K
1580	80	R.DGEDQTQDTELVETRPAGDGTFQK.W

GRP78_HUMAN Mass: 72332 Score: 108 Matches: 5(3) Sequences: 4(3) emPAI: 0.18
78 kDa glucose-regulated protein OS=Homo sapiens GN=HSPA5 PE=1 SV=2

Query	Score	Peptide
729	-38	R.VEIIANDQGNR.I
730	46	R.VEIIANDQGNR.I
839	38	R.NELESYAYSLK.N
922	53	R.TWNDPSVQQDIK.F
1094	84	K.NQLTSNPENTVFDAK.R

H2B1C_HUMAN Mass: 13906 Score: 106 Matches: 2(2) Sequences: 2(2) emPAI: 0.76
Histone H2B type 1-C/E/F/G/I OS=Homo sapiens GN=HIST1H2BC PE=1 SV=4

Query	Score	Peptide
780	39	R.KESYSVYVYK.V
1208	104	K.AMGIMNSFVNDIFER.I + 2 Oxidation (M)

CD44_HUMAN Mass: 81537 Score: 99 Matches: 2(2) Sequences: 2(2) emPAI: 0.11
CD44 antigen OS=Homo sapiens GN=CD44 PE=1 SV=3

Query	Score	Peptide
875	60	K.LVINSGNGAVEDR.K
1271	77	K.ESSETPDQFMTADETR.N

MFGM_HUMAN Mass: 43122 Score: 91 Matches: 1(1) Sequences: 1(1) emPAI: 0.10
Lactadherin OS=Homo sapiens GN=MFGE8 PE=1 SV=2

Query	Score	Peptide
619	91	K.EVTGIITQGAR.N

LDHA_HUMAN Mass: 36688 Score: 81 Matches: 3(3) Sequences: 3(3) emPAI: 0.39
L-lactate dehydrogenase A chain OS=Homo sapiens GN=LDHA PE=1 SV=2

Query	Score	Peptide
591	53	K.SADTLWGIQK.E

609	44	K.VTLTSEEEAR.L
778	55	K.QVVESAYEVIK.L

K22E_HUMAN Mass: 65432 Score: 78 Matches: 1(1) Sequences: 1(1) emPAI: 0.06
Keratin, type II cytoskeletal 2 epidermal OS=Homo sapiens GN=KRT2 PE=1 SV=2

Query	Score	Peptide
934	78	R.FLEQQNQVLQTK.W

EDIL3_HUMAN Mass: 53764 Score: 77 Matches: 2(1) Sequences: 2(1) emPAI: 0.08
EGF-like repeat and discoidin I-like domain-containing protein 3 OS=Homo sapiens GN=EDIL3 PE=1 SV=1

Query	Score	Peptide
645	36	K.NVIDPPIYAR.H
1383	77	R.GNIDNNTPYANSFTPPIK.A

PGK1_HUMAN Mass: 44614 Score: 69 Matches: 2(2) Sequences: 2(2) emPAI: 0.20
Phosphoglycerate kinase 1 OS=Homo sapiens GN=PGK1 PE=1 SV=3

Query	Score	Peptide
577	53	K.VLPGVDALSNI.-
892	49	R.AHSSMVGVNLPQK.A

CH10_HUMAN Mass: 10932 Score: 67 Matches: 1(1) Sequences: 1(1) emPAI: 0.43
10 kDa heat shock protein, mitochondrial OS=Homo sapiens GN=HSPE1 PE=1 SV=2

Query	Score	Peptide
836	67	K.VLQATVVAVGSGSK.G

MYOF_HUMAN Mass: 234706 Score: 65 Matches: 1(1) Sequences: 1(1) emPAI: 0.02
Myoferlin OS=Homo sapiens GN=MYOF PE=1 SV=1

Query	Score	Peptide
798	65	K.GPVGTVSEAQLAR.R

GTR1_HUMAN Mass: 54083 Score: 61 Matches: 1(1) Sequences: 1(1) emPAI: 0.08
Solute carrier family 2, facilitated glucose transporter member 1 OS=Homo sapiens GN=SLC2A1 PE=1 SV=2

Query	Score	Peptide
617	61	R.TFDEIASGFR.Q

BASI_HUMAN Mass: 42200 Score: 59 Matches: 2(2) Sequences: 2(2) emPAI: 0.21
Basigin OS=Homo sapiens GN=BSG PE=1 SV=2

Query	Score	Peptide
510	39	R.FFVSSSQGR.S
915	54	K.GGVVLKEDALPGQK.T

MYH9_HUMAN Mass: 226530 Score: 58 Matches: 1(1) Sequences: 1(1) emPAI: 0.02
Myosin-9 OS=Homo sapiens GN=MYH9 PE=1 SV=4

Query	Score	Peptide

683	58	K.ALELDSNLYR.I

COF1_HUMAN Mass: 18502 Score: 57 Matches: 1(1) Sequences: 1(1) emPAI: 0.24
Cofilin-1 OS=Homo sapiens GN=CFL1 PE=1 SV=3

Query	Score	Peptide
869	57	R.YALYDATYETK.E

HMGA1_HUMAN Mass: 11676 Score: 57 Matches: 3(2) Sequences: 2(2) emPAI: 1.72
High mobility group protein HMG-I/HMG-Y OS=Homo sapiens GN=HMGA1 PE=1 SV=3

Query	Score	Peptide
1040	47	R.KQPPVSPGTALVGSQK.E
1511	-36	K.KLEKEEEEGISQESSEEEQ.-
1512	42	K.KLEKEEEEGISQESSEEEQ.-

PPIA_HUMAN Mass: 18012 Score: 56 Matches: 1(1) Sequences: 1(1) emPAI: 0.25
Peptidyl-prolyl cis-trans isomerase A OS=Homo sapiens GN=PPIA PE=1 SV=2

Query	Score	Peptide
638	56	K.FEDENFILK.H

MYL6B_HUMAN Mass: 22764 Score: 56 Matches: 1(1) Sequences: 1(1) emPAI: 0.19
Myosin light chain 6B OS=Homo sapiens GN=MYL6B PE=1 SV=1

Query	Score	Peptide
882	56	R.ALGQNPTNAEVLK.V

S10AA_HUMAN Mass: 11203 Score: 50 Matches: 1(1) Sequences: 1(1) emPAI: 0.41
Protein S100-A10 OS=Homo sapiens GN=S100A10 PE=1 SV=2

Query	Score	Peptide
1357	50	K.EFPGFLENQKDPLAVDK.I

PLP2_HUMAN Mass: 16691 Score: 48 Matches: 1(1) Sequences: 1(1) emPAI: 0.27
Proteolipid protein 2 OS=Homo sapiens GN=PLP2 PE=1 SV=1

Query	Score	Peptide
763	48	R.HTAAPTDPADGPV.-

THIO_HUMAN Mass: 11737 Score: 47 Matches: 1(1) Sequences: 1(1) emPAI: 0.39
Thioredoxin OS=Homo sapiens GN=TXN PE=1 SV=3

Query	Score	Peptide
864	47	K.TAFQEALDAAGDK.L

LMNA_HUMAN Mass: 74139 Score: 43 Matches: 1(1) Sequences: 1(1) emPAI: 0.06
Prelamin-A/C OS=Homo sapiens GN=LMNA PE=1 SV=1

Query	Score	Peptide
651	43	K.AAYEAELGDAR.K

RL7_HUMAN Mass: 29225 Score: 41 Matches: 1(1) Sequences: 1(1) emPAI: 0.15

60S ribosomal protein L7 OS=Homo sapiens GN=RPL7 PE=1 SV=1

Query	Score	Peptide
659	41	R.IALTDNALIAR.S

GNAO_HUMAN Mass: 40050 Score: 41 Matches: 1(1) Sequences: 1(1) emPAI: 0.11

Guanine nucleotide-binding protein G(o) subunit alpha OS=Homo sapiens GN=GNAO1 PE=1 SV=4

Query	Score	Peptide
900	41	K.TTGIVETHFTFK.N

AHNK_HUMAN Mass: 629094 Score: 41 Matches: 1(1) Sequences: 1(1) emPAI: 0.01

Neuroblast differentiation-associated protein AHNAK OS=Homo sapiens GN=AHNAK PE=1 SV=2

Query	Score	Peptide
767	41	K.GEGPEVDVNLPK.A

TCPH_HUMAN Mass: 59366 Score: 39 Matches: 1(1) Sequences: 1(1) emPAI: 0.07

T-complex protein 1 subunit eta OS=Homo sapiens GN=CCT7 PE=1 SV=2

Query	Score	Peptide
697	39	K.ATISNDGATILK.L

TMSL3_HUMAN Mass: 5063 Score: 39 Matches: 1(1) Sequences: 1(1) emPAI: 1.03

Thymosin beta-4-like protein 3 OS=Homo sapiens GN=TMSL3 PE=2 SV=1

Query	Score	Peptide
964	39	K.NPLPSKETIEQEK.Q
