# Supplementary material for: Molecular characterization of exosome-like vesicles from breast cancer cells
Source: BMC Cancer. 2014 Jan 27;14:44. doi: 10.1186/1471-2407-14-44 (PMC3936808; doi:10.1186/1471-2407-14-44)
Supplement: Additional file 2: Table S2 — List of proteins exclusively identified in MDA-Exo. [file 1471-2407-14-44-S2.pdf]

Supplementary Table 2: List of proteins exclusively identified in MDA-Exo.

**ACTB_HUMAN** Mass: 41736 Score: 1424 Matches: 53(49) Sequences: 12(12) emPAI: 5.33
Actin, cytoplasmic 1 OS=Homo sapiens GN=ACTB PE=1 SV=1

| Query | Score | Peptide |
|---|---|---|
| 364 | 47 | R.DLTDYLMK.I |
| 395 | -46 | R.DLTDYLMK.I + Oxidation (M) |
| 528 | -45 | R.GYSFTTTAER.E |
| 529 | 74 | R.GYSFTTTAER.E |
| 530 | -49 | R.GYSFTTTAER.E |
| 531 | -74 | R.GYSFTTTAER.E |
| 532 | -46 | R.GYSFTTTAER.E |
| 533 | -48 | R.GYSFTTTAER.E |
| 534 | -51 | R.GYSFTTTAER.E |
| 536 | -35 | R.GYSFTTTAER.E |
| 537 | -54 | R.GYSFTTTAER.E |
| 538 | -37 | R.GYSFTTTAER.E |
| 539 | -50 | R.GYSFTTTAER.E |
| 540 | -49 | R.GYSFTTTAER.E |
| 541 | -36 | R.GYSFTTTAER.E |
| 542 | -71 | R.GYSFTTTAER.E |
| 592 | -52 | K.EITALAPSTMK.I |
| 593 | 81 | K.EITALAPSTMK.I |
| 594 | -39 | K.EITALAPSTMK.I |
| 595 | -42 | K.EITALAPSTMK.I |
| 610 | -47 | R.HQGVMVGMGQK.D |
| 611 | 48 | R.HQGVMVGMGQK.D |
| 612 | -35 | R.HQGVMVGMGQK.D |
| 629 | -60 | K.EITALAPSTMK.I + Oxidation (M) |
| 630 | -43 | K.EITALAPSTMK.I + Oxidation (M) |
| 631 | -40 | K.EITALAPSTMK.I + Oxidation (M) |
| 654 | 48 | R.AVFPSIVGRPR.H |
| 983 | -56 | K.QEYDESGPSIVHR.K + Gln->pyro-Glu (N-term Q) |
| 1001 | 44 | K.IWHHTFYNELR.V |
| 1003 | -40 | K.IWHHTFYNELR.V |
| 1004 | -52 | K.QEYDESGPSIVHR.K |
| 1008 | 57 | K.QEYDESGPSIVHR.K |
| 1010 | -45 | K.QEYDESGPSIVHR.K |
| 1015 | -44 | K.QEYDESGPSIVHR.K |
| 1362 | -99 | K.SYELPDGQVITIGNER.F |
| 1363 | 118 | K.SYELPDGQVITIGNER.F |
| 1364 | -109 | K.SYELPDGQVITIGNER.F |
| 1365 | -72 | K.SYELPDGQVITIGNER.F |
| 1366 | -63 | K.SYELPDGQVITIGNER.F |
| 1367 | -89 | K.SYELPDGQVITIGNER.F |
| 1368 | -83 | K.SYELPDGQVITIGNER.F |
| 1537 | -64 | R.VAPEEHPVLLTEAPLNPK.A |
| 1538 | 93 | R.VAPEEHPVLLTEAPLNPK.A |
| 1539 | -52 | R.VAPEEHPVLLTEAPLNPK.A |
| 1542 | -65 | R.VAPEEHPVLLTEAPLNPK.A |
| 1545 | -42 | R.VAPEEHPVLLTEAPLNPK.A |
| 1547 | -50 | R.VAPEEHPVLLTEAPLNPK.A |
| 1734 | -104 | K.DLYANTVLSGGTTMYPGIADR.M |

| | | |
|---|---|---|
| 1746 | 117 | K.DLYANTVLSGGTTMYPGIADR.M + Oxidation (M) |
| 1780 | 134 | R.KDLYANTVLSGGTTMYPGIADR.M |
| 1781 | -81 | R.KDLYANTVLSGGTTMYPGIADR.M |
| 1895 | 102 | R.TTGIVMDSGDGVTHTVPIYEGYALPHAILR.L |
| 1896 | -52 | R.TTGIVMDSGDGVTHTVPIYEGYALPHAILR.L |

**ACTC_HUMAN** Mass: 42019 Score: 644 Matches: 26(25) Sequences: 7(7) emPAI: 1.62
Actin, alpha cardiac muscle 1 OS=Homo sapiens GN=ACTC1 PE=1 SV=1

| Query | Score | Peptide |
|---|---|---|
| 364 | 47 | R.DLTDYLMK.I |
| 395 | -46 | R.DLTDYLMK.I + Oxidation (M) |
| 592 | -52 | K.EITALAPSTMK.I |
| 593 | 81 | K.EITALAPSTMK.I |
| 594 | -39 | K.EITALAPSTMK.I |
| 595 | -42 | K.EITALAPSTMK.I |
| 610 | -47 | R.HQGVMVGMGQK.D |
| 611 | 48 | R.HQGVMVGMGQK.D |
| 612 | -35 | R.HQGVMVGMGQK.D |
| 629 | -60 | K.EITALAPSTMK.I + Oxidation (M) |
| 630 | -43 | K.EITALAPSTMK.I + Oxidation (M) |
| 631 | -40 | K.EITALAPSTMK.I + Oxidation (M) |
| 654 | 48 | R.AVFPSIVGRPR.H |
| 1001 | 44 | K.IWHHTFYNELR.V |
| 1003 | -40 | K.IWHHTFYNELR.V |
| 1362 | -99 | K.SYELPDGQVITIGNER.F |
| 1363 | 118 | K.SYELPDGQVITIGNER.F |
| 1364 | -109 | K.SYELPDGQVITIGNER.F |
| 1365 | -72 | K.SYELPDGQVITIGNER.F |
| 1366 | -63 | K.SYELPDGQVITIGNER.F |
| 1367 | -89 | K.SYELPDGQVITIGNER.F |
| 1368 | -83 | K.SYELPDGQVITIGNER.F |
| 1561 | -41 | K.YPIEHGIITNWDDMEK.I |
| 1562 | 63 | K.YPIEHGIITNWDDMEK.I |
| 1563 | -44 | K.YPIEHGIITNWDDMEK.I |
| 1589 | -50 | K.YPIEHGIITNWDDMEK.I + Oxidation (M) |

**TBB2C_HUMAN** Mass: 49830 Score: 381 Matches: 10(10) Sequences: 7(7) emPAI: 1.08
Tubulin beta-2C chain OS=Homo sapiens GN=TUBB2C PE=1 SV=1

| Query | Score | Peptide |
|---|---|---|
| 556 | 65 | K.LAVNMVPFPR.L |
| 839 | -83 | R.IMNTFSVVPSPK.V |
| 845 | 91 | R.IMNTFSVVPSPK.V |
| 872 | -46 | R.IMNTFSVVPSPK.V + Oxidation (M) |
| 954 | 75 | K.EVDEQMLNVQNK.N |
| 960 | -63 | K.EVDEQMLNVQNK.N + Oxidation (M) |
| 1129 | 77 | R.AVLVDLEPGTMDSVR.S |
| 1204 | 92 | R.ALTVPELTQQMFDAK.N |
| 1209 | 74 | K.NSSYFVEWIPNNVK.T |
| 1720 | 53 | R.GRMSMKEVDEQMLNVQNK.N + 2 Oxidation (M) |

**HSP71_HUMAN** Mass: 70051 Score: 284 Matches: 9(8) Sequences: 7(7) emPAI: 0.50

Heat shock 70 kDa protein 1A/1B OS=Homo sapiens GN=HSPA1A PE=1 SV=5

| Query | Score | Peptide |
|---|---|---|
| 653 | 47 | K.DAGVIAGLNVLR.I |
| 697 | 44 | K.FGDPVVQSDMK.H |
| 700 | -36 | K.VEIIANDQGNR.T |
| 701 | -41 | K.VEIIANDQGNR.T |
| 703 | 44 | K.VEIIANDQGNR.T |
| 976 | 103 | R.TTPSYVAFTDTER.L |
| 1174 | 93 | K.NQVALNPQNTVFDAK.R |
| 1186 | 78 | K.ATAGDTHLGGEDFDNR.L |
| 1199 | 88 | R.IINEPTAAAIAYGLDR.T |

**TBB3_HUMAN** Mass: 50432 Score: 279 Matches: 7(7) Sequences: 5(5) emPAI: 0.62
Tubulin beta-3 chain OS=Homo sapiens GN=TUBB3 PE=1 SV=2

| Query | Score | Peptide |
|---|---|---|
| 556 | 65 | K.LAVNMVPFPR.L |
| 839 | -83 | R.IMNTFSVVPSPK.V |
| 845 | 91 | R.IMNTFSVVPSPK.V |
| 872 | -46 | R.IMNTFSVVPSPK.V + Oxidation (M) |
| 925 | 55 | K.EVDEQMLAIQSK.N |
| 1204 | 92 | R.ALTVPELTQQMFDAK.N |
| 1209 | 74 | K.NSSYFVEWIPNNVK.V |

**CLH1_HUMAN** Mass: 191612 Score: 195 Matches: 7(7) Sequences: 7(7) emPAI: 0.16
Clathrin heavy chain 1 OS=Homo sapiens GN=CLTC PE=1 SV=5

| Query | Score | Peptide |
|---|---|---|
| 720 | 47 | K.TLQIFNIEMK.S |
| 880 | 42 | R.VVGAMQLYSVDR.K |
| 1085 | 72 | R.RPISADSAIMNPASK.V |
| 1245 | 84 | K.VSQPIEGHAASFAQFK.M |
| 1529 | 69 | K.LHIIEVGTPPTGNQPFPK.K |
| 1779 | 55 | R.RPISADSAIMNPASKVIALKAGK.T |
| 1859 | 41 | K.ADDPSSYMEVVQAANTSGNWEELVK.Y |

**UGDH_HUMAN** Mass: 55023 Score: 176 Matches: 5(5) Sequences: 5(5) emPAI: 0.45
UDP-glucose 6-dehydrogenase OS=Homo sapiens GN=UGDH PE=1 SV=1

| Query | Score | Peptide |
|---|---|---|
| 471 | 79 | K.LAANAFLAQR.I |
| 510 | 39 | R.VTVVDVNESR.I |
| 914 | 43 | R.VLIGGDETPEGQR.A |
| 971 | 86 | K.ILTTNTWSSELSK.L |
| 1500 | 73 | R.INAWNSPTLPIYEPGLK.E |

**K2C8_HUMAN** Mass: 53704 Score: 149 Matches: 7(7) Sequences: 7(7) emPAI: 0.70
Keratin, type II cytoskeletal 8 OS=Homo sapiens GN=KRT8 PE=1 SV=7

| Query | Score | Peptide |
|---|---|---|
| 366 | 53 | R.LQAEIEGLK.G |
| 475 | 39 | R.QLYEEEIR.E |
| 580 | 39 | R.EYQELMNVK.L |
| 619 | 49 | K.LVSESSDVLPK.- |

| Query | Score | Peptide |
|---|---|---|
| 893 | 57 | R.ASLEAAIADAEQR.G |
| 897 | 67 | R.TEMENEFVLIK.K |
| 940 | 69 | R.LEGLTDEINFLR.Q |

**H2B1C_HUMAN** Mass: 13906 Score: 139 Matches: 5(3) Sequences: 4(3) emPAI: 2.10
Histone H2B type 1-C/E/F/G/I OS=Homo sapiens GN=HIST1H2BC PE=1 SV=4

| Query | Score | Peptide |
|---|---|---|
| 545 | 36 | K.ESYSVYVYK.V |
| 573 | 50 | K.QVHPDTGISSK.A + Gln->pyro-Glu (N-term Q) |
| 578 | -35 | K.QVHPDTGISSK.A + Gln->pyro-Glu (N-term Q) |
| 757 | 62 | R.KESYSVYVYK.V |
| 1350 | 96 | K.AMGIMNSFVNDIFER.I + 2 Oxidation (M) |

**AT1A3_HUMAN** Mass: 111747 Score: 125 Matches: 5(5) Sequences: 5(5) emPAI: 0.20
Sodium/potassium-transporting ATPase subunit alpha-3 OS=Homo sapiens GN=ATP1A3 PE=1 SV=3

| Query | Score | Peptide |
|---|---|---|
| 719 | 50 | R.LNIPVSQVNPR.D |
| 922 | 48 | K.VIMVTGDHPITAK.A |
| 1140 | 69 | K.VDNSSLTGESEPQTR.S |
| 1421 | 53 | K.GVGIISEGNETVEDIAAR.L |
| 1798 | 53 | R.IISAHGCKVDNSSLTGESEPQTR.S |

**EPCAM_HUMAN** Mass: 34932 Score: 118 Matches: 1(1) Sequences: 1(1) emPAI: 0.12
Epithelial cell adhesion molecule OS=Homo sapiens GN=EPCAM PE=1 SV=2

| Query | Score | Peptide |
|---|---|---|
| 1489 | 118 | K.TQNDVDIADVAYYFEK.D |

**STOM_HUMAN** Mass: 31730 Score: 115 Matches: 1(1) Sequences: 1(1) emPAI: 0.14
Erythrocyte band 7 integral membrane protein OS=Homo sapiens GN=STOM PE=1 SV=3

| Query | Score | Peptide |
|---|---|---|
| 1514 | 115 | R.VQNATLAVANITNADSATR.L |

**4F2_HUMAN** Mass: 67993 Score: 109 Matches: 3(2) Sequences: 3(2) emPAI: 0.13
4F2 cell-surface antigen heavy chain OS=Homo sapiens GN=SLC3A2 PE=1 SV=3

| Query | Score | Peptide |
|---|---|---|
| 607 | 37 | K.ADLLLSTQPGR.E |
| 977 | 70 | K.IKVAEDEAEAAAAAK.F |
| 991 | 70 | K.GQSEDPGSLLSLFR.R |

**GNAI3_HUMAN** Mass: 40532 Score: 109 Matches: 4(4) Sequences: 4(4) emPAI: 0.49
Guanine nucleotide-binding protein G(k) subunit alpha OS=Homo sapiens GN=GNAI3 PE=1 SV=3

| Query | Score | Peptide |
|---|---|---|
| 447 | 69 | K.LLLLGAGESGK.S |
| 921 | 57 | K.TTGIVETHFTFK.D |
| 1100 | 40 | K.LLLLGAGESGKSTIVK.Q |
| 1325 | 52 | R.ISQSNYIPTQQDVLR.T |

**ALDOA_HUMAN** Mass: 39420 Score: 104 Matches: 2(2) Sequences: 2(2) emPAI: 0.23

Fructose-bisphosphate aldolase A OS=Homo sapiens GN=ALDOA PE=1 SV=2

| Query | Score | Peptide |
|---|---|---|
| 870 | 85 | K.GILAADESTGSIAK.R |
| 947 | 54 | M.PYQYPALTPEQK.K |

**RS3_HUMAN** Mass: 26688 Score: 104 Matches: 1(1) Sequences: 1(1) emPAI: 0.16
40S ribosomal protein S3 OS=Homo sapiens GN=RPS3 PE=1 SV=2

| Query | Score | Peptide |
|---|---|---|
| 943 | 104 | R.ELAEDGYSGVEVR.V |

**RS8_HUMAN** Mass: 24205 Score: 103 Matches: 2(2) Sequences: 2(2) emPAI: 0.39
40S ribosomal protein S8 OS=Homo sapiens GN=RPS8 PE=1 SV=2

| Query | Score | Peptide |
|---|---|---|
| 827 | 54 | K.LTPEEEEILNK.K |
| 1251 | 87 | R.IIDVVYNASNNELVR.T |

**RAI3_HUMAN** Mass: 40251 Score: 100 Matches: 1(1) Sequences: 1(1) emPAI: 0.11
Retinoic acid-induced protein 3 OS=Homo sapiens GN=GPRC5A PE=1 SV=2

| Query | Score | Peptide |
|---|---|---|
| 946 | 100 | R.TNVNVFSELSAPR.R |

**HSPB1_HUMAN** Mass: 22782 Score: 99 Matches: 3(2) Sequences: 3(2) emPAI: 0.42
Heat shock protein beta-1 OS=Homo sapiens GN=HSPB1 PE=1 SV=2

| Query | Score | Peptide |
|---|---|---|
| 448 | 36 | R.QLSSGVSEIR.H + Gln->pyro-Glu (N-term Q) |
| 596 | 66 | R.LFDQAFGLPR.L |
| 1496 | 71 | K.LATQSNEITIPVTFESR.A |

**K2C1_HUMAN** Mass: 66038 Score: 95 Matches: 2(2) Sequences: 2(2) emPAI: 0.13
Keratin, type II cytoskeletal 1 OS=Homo sapiens GN=KRT1 PE=1 SV=6

| Query | Score | Peptide |
|---|---|---|
| 758 | 43 | R.TNAENEFVTIK.K |
| 964 | 90 | R.FLEQQNQVLQTK.W |

**GNAS1_HUMAN** Mass: 111023 Score: 90 Matches: 3(3) Sequences: 3(3) emPAI: 0.12
Guanine nucleotide-binding protein G(s) subunit alpha isoforms XLas OS=Homo sapiens GN=GNAS PE=1 SV=2

| Query | Score | Peptide |
|---|---|---|
| 447 | 69 | R.LLLLGAGESGK.S |
| 1100 | 40 | R.LLLLGAGESGKSTIVK.Q |
| 1349 | 48 | R.YTTPEDATPEPGEDPR.V |

**PPIA_HUMAN** Mass: 18012 Score: 89 Matches: 2(2) Sequences: 2(2) emPAI: 0.55
Peptidyl-prolyl cis-trans isomerase A OS=Homo sapiens GN=PPIA PE=1 SV=2

| Query | Score | Peptide |
|---|---|---|
| 581 | 39 | K.FEDENFILK.H |
| 992 | 87 | K.VKEGMNIVEAMER.F |

**UBA1_HUMAN** Mass: 117848 Score: 86 Matches: 2(2) Sequences: 2(2) emPAI: 0.07
Ubiquitin-like modifier-activating enzyme 1 OS=Homo sapiens GN=UBA1 PE=1 SV=3

| Query | Score | Peptide |
|---|---|---|
| 731 | 45 | R.KPLLESGTLGTK.G |
| 1484 | 78 | R.NEEDAAELVALAQAVNAR.A |

**RSSA_HUMAN** Mass: 32854 Score: 85 Matches: 1(1) Sequences: 1(1) emPAI: 0.13
40S ribosomal protein SA OS=Homo sapiens GN=RPSA PE=1 SV=4

| Query | Score | Peptide |
|---|---|---|
| 671 | 85 | K.FAAATGATPIAGR.F |

**NDKA_HUMAN** Mass: 17149 Score: 83 Matches: 1(1) Sequences: 1(1) emPAI: 0.26
Nucleoside diphosphate kinase A OS=Homo sapiens GN=NME1 PE=1 SV=1

| Query | Score | Peptide |
|---|---|---|
| 1355 | 83 | R.VMLGETNPADSKPGTIR.G |

**HNRPK_HUMAN** Mass: 50976 Score: 82 Matches: 1(1) Sequences: 1(1) emPAI: 0.08
Heterogeneous nuclear ribonucleoprotein K OS=Homo sapiens GN=HNRNPK PE=1 SV=1

| Query | Score | Peptide |
|---|---|---|
| 1352 | 82 | R.TDYNASVSVPDSSGPER.I |

**ARF1_HUMAN** Mass: 20697 Score: 82 Matches: 1(1) Sequences: 1(1) emPAI: 0.21
ADP-ribosylation factor 1 OS=Homo sapiens GN=ARF1 PE=1 SV=2

| Query | Score | Peptide |
|---|---|---|
| 1152 | 82 | K.QDLPNAMNAAEITDK.L |

**RL7A_HUMAN** Mass: 29995 Score: 78 Matches: 1(1) Sequences: 1(1) emPAI: 0.14
60S ribosomal protein L7a OS=Homo sapiens GN=RPL7A PE=1 SV=2

| Query | Score | Peptide |
|---|---|---|
| 894 | 78 | R.AGVNTVTTLVENK.K |

**K1C18_HUMAN** Mass: 48057 Score: 73 Matches: 1(1) Sequences: 1(1) emPAI: 0.09
Keratin, type I cytoskeletal 18 OS=Homo sapiens GN=KRT18 PE=1 SV=2

| Query | Score | Peptide |
|---|---|---|
| 941 | 73 | R.QAQEYEALLNIK.V |

**RAN_HUMAN** Mass: 24423 Score: 71 Matches: 2(2) Sequences: 2(2) emPAI: 0.39
GTP-binding nuclear protein Ran OS=Homo sapiens GN=RAN PE=1 SV=3

| Query | Score | Peptide |
|---|---|---|
| 686 | 51 | K.NLQYYDISAK.S |
| 780 | 57 | K.FNVWDTAGQEK.F |

**AAAT_HUMAN** Mass: 56598 Score: 70 Matches: 2(2) Sequences: 2(2) emPAI: 0.15
Neutral amino acid transporter B(0) OS=Homo sapiens GN=SLC1A5 PE=1 SV=2

| Query | Score | Peptide |
|---|---|---|

| Query | Score | Peptide |
|---|---|---|
| 557 | 55 | R.STEPELIQVK.S |
| 1717 | 51 | K.SELPLDPLPVPTEEGNPLLK.H |

**RS2_HUMAN** Mass: 31324 Score: 70 Matches: 2(1) Sequences: 2(1) emPAI: 0.14
40S ribosomal protein S2 OS=Homo sapiens GN=RPS2 PE=1 SV=2

| Query | Score | Peptide |
|---|---|---|
| 414 | 36 | R.GTGIVSAPVPK.K |
| 961 | 70 | K.SPYQEFTDHLVK.T |

**LAT1_HUMAN** Mass: 55010 Score: 69 Matches: 1(1) Sequences: 1(1) emPAI: 0.08
Large neutral amino acids transporter small subunit 1 OS=Homo sapiens GN=SLC7A5 PE=1 SV=2

| Query | Score | Peptide |
|---|---|---|
| 1226 | 69 | K.SADGSAPAGEGEGVTLQR.N |

**RAB1A_HUMAN** Mass: 22677 Score: 68 Matches: 1(1) Sequences: 1(1) emPAI: 0.19
Ras-related protein Rab-1A OS=Homo sapiens GN=RAB1A PE=1 SV=3

| Query | Score | Peptide |
|---|---|---|
| 830 | 68 | K.LQIWDTAGQER.F |

**RS26_HUMAN** Mass: 13015 Score: 68 Matches: 1(1) Sequences: 1(1) emPAI: 0.35
40S ribosomal protein S26 OS=Homo sapiens GN=RPS26 PE=1 SV=3

| Query | Score | Peptide |
|---|---|---|
| 1171 | 68 | R.DISEASVFDAYVLPK.L |

**RS24_HUMAN** Mass: 15423 Score: 65 Matches: 1(1) Sequences: 1(1) emPAI: 0.29
40S ribosomal protein S24 OS=Homo sapiens GN=RPS24 PE=1 SV=1

| Query | Score | Peptide |
|---|---|---|
| 1195 | 65 | K.TTGFGMIYDSLDYAK.K |

**1A01_HUMAN** Mass: 40845 Score: 64 Matches: 1(1) Sequences: 1(1) emPAI: 0.10
HLA class I histocompatibility antigen, A-1 alpha chain OS=Homo sapiens GN=HLA-A PE=1 SV=1

| Query | Score | Peptide |
|---|---|---|
| 1837 | 64 | R.DGEDQTQDTELVETRPAGDGTFQK.W |

**CLIC1_HUMAN** Mass: 26922 Score: 59 Matches: 1(1) Sequences: 1(1) emPAI: 0.16
Chloride intracellular channel protein 1 OS=Homo sapiens GN=CLIC1 PE=1 SV=4

| Query | Score | Peptide |
|---|---|---|
| 769 | 59 | K.GVTFNVTTVDTK.R |

**K6PP_HUMAN** Mass: 85595 Score: 57 Matches: 1(1) Sequences: 1(1) emPAI: 0.05
6-phosphofructokinase type C OS=Homo sapiens GN=PFKP PE=1 SV=2

| Query | Score | Peptide |
|---|---|---|
| 939 | 57 | R.DLQSNVEHLTEK.M |

**RS6_HUMAN** Mass: 28680 Score: 55 Matches: 1(1) Sequences: 1(1) emPAI: 0.15
40S ribosomal protein S6 OS=Homo sapiens GN=RPS6 PE=1 SV=1

| Query | Score | Peptide |
|---|---|---|
| 770 | 55 | K.DIPGLTDTTVPR.R |

**LEG3_HUMAN** Mass: 26152 Score: 54 Matches: 1(1) Sequences: 1(1) emPAI: 0.17
Galectin-3 OS=Homo sapiens GN=LGALS3 PE=1 SV=5

| Query | Score | Peptide |
|---|---|---|
| 761 | 54 | R.GNDVAFHFNPR.F |

**ANXA6_HUMAN** Mass: 75872 Score: 52 Matches: 2(2) Sequences: 2(2) emPAI: 0.11
Annexin A6 OS=Homo sapiens GN=ANXA6 PE=1 SV=3

| Query | Score | Peptide |
|---|---|---|
| 501 | 41 | R.SELDMLDIR.E + Oxidation (M) |
| 626 | 49 | R.DAFVAIVQSVK.N |

**RL17_HUMAN** Mass: 21397 Score: 51 Matches: 1(1) Sequences: 1(1) emPAI: 0.20
60S ribosomal protein L17 OS=Homo sapiens GN=RPL17 PE=1 SV=3

| Query | Score | Peptide |
|---|---|---|
| 1145 | 51 | K.EQIVPKPEEEVAQK.K |

**RL3_HUMAN** Mass: 46108 Score: 50 Matches: 1(1) Sequences: 1(1) emPAI: 0.09
60S ribosomal protein L3 OS=Homo sapiens GN=RPL3 PE=1 SV=2

| Query | Score | Peptide |
|---|---|---|
| 890 | 50 | K.NNASTDYDLSDK.S |

**MUC1_HUMAN** Mass: 122101 Score: 49 Matches: 1(1) Sequences: 1(1) emPAI: 0.03
Mucin-1 OS=Homo sapiens GN=MUC1 PE=1 SV=3

| Query | Score | Peptide |
|---|---|---|
| 779 | 49 | K.NYGQLDIFPAR.D |

**PUR9_HUMAN** Mass: 64615 Score: 49 Matches: 1(1) Sequences: 1(1) emPAI: 0.07
Bifunctional purine biosynthesis protein PURH OS=Homo sapiens GN=ATIC PE=1 SV=3

| Query | Score | Peptide |
|---|---|---|
| 1130 | 49 | R.DVSELTGFPEMLGGR.V |

**RS16_HUMAN** Mass: 16445 Score: 49 Matches: 1(1) Sequences: 1(1) emPAI: 0.27
40S ribosomal protein S16 OS=Homo sapiens GN=RPS16 PE=1 SV=2

| Query | Score | Peptide |
|---|---|---|
| 495 | 49 | K.LLEPVLLLGK.E |

**DDX5_HUMAN** Mass: 69147 Score: 47 Matches: 1(1) Sequences: 1(1) emPAI: 0.06
Probable ATP-dependent RNA helicase DDX5 OS=Homo sapiens GN=DDX5 PE=1 SV=1

| Query | Score | Peptide |
|---|---|---|
| 699 | 47 | K.APILIATDVASR.G |

**K1C19_HUMAN** Mass: 44105 Score: 46 Matches: 1(1) Sequences: 1(1) emPAI: 0.10
Keratin, type I cytoskeletal 19 OS=Homo sapiens GN=KRT19 PE=1 SV=4

| Query | Score | Peptide |
|---|---|---|
| 730 | 46 | R.ALEAANGELEVK.I |

**PSD1_HUMAN** Mass: 109541 Score: 45 Matches: 1(1) Sequences: 1(1) emPAI: 0.04
PH and SEC7 domain-containing protein 1 OS=Homo sapiens GN=PSD PE=2 SV=2

| Query | Score | Peptide |
|---|---|---|
| 850 | 45 | R.RSLSELADPNPK.V |

**EZRI_HUMAN** Mass: 69412 Score: 42 Matches: 1(1) Sequences: 1(1) emPAI: 0.06
Ezrin OS=Homo sapiens GN=EZR PE=1 SV=4

| Query | Score | Peptide |
|---|---|---|
| 500 | 42 | K.IGFPWSEIR.N |

**H2A1B_HUMAN** Mass: 14135 Score: 42 Matches: 1(1) Sequences: 1(1) emPAI: 0.32
Histone H2A type 1-B/E OS=Homo sapiens GN=HIST1H2AB PE=1 SV=2

| Query | Score | Peptide |
|---|---|---|
| 1516 | 42 | R.VTIAQGGVLPNIQAVLLPK.K |

**RNF17_HUMAN** Mass: 184641 Score: 41 Matches: 1(1) Sequences: 1(1) emPAI: 0.02
RING finger protein 17 OS=Homo sapiens GN=RNF17 PE=1 SV=3

| Query | Score | Peptide |
|---|---|---|
| 863 | 41 | K.KNNLNAAMNIAR.A |

**AT1B1_HUMAN** Mass: 35061 Score: 40 Matches: 1(1) Sequences: 1(1) emPAI: 0.12
Sodium/potassium-transporting ATPase subunit beta-1 OS=Homo sapiens GN=ATP1B1 PE=1 SV=1

| Query | Score | Peptide |
|---|---|---|
| 978 | 40 | R.VAPPGLTQIPQIQK.T |

**PGK1_HUMAN** Mass: 44614 Score: 40 Matches: 1(1) Sequences: 1(1) emPAI: 0.10
Phosphoglycerate kinase 1 OS=Homo sapiens GN=PGK1 PE=1 SV=3

| Query | Score | Peptide |
|---|---|---|
| 1160 | 40 | K.LGDVYVNDAFGTAHR.A |

Proteins matching the same set of peptides:
PGK2_HUMAN Mass: 44796 Score: 40 Matches: 1(1) Sequences: 1(1)
Phosphoglycerate kinase 2 OS=Homo sapiens GN=PGK2 PE=1 SV=3

**NQO1_HUMAN** Mass: 30867 Score: 40 Matches: 1(1) Sequences: 1(1) emPAI: 0.14
NAD(P)H dehydrogenase [quinone] 1 OS=Homo sapiens GN=NQO1 PE=1 SV=1

| Query | Score | Peptide |
|---|---|---|
| 503 | 40 | R.ALIVLAHSER.T |

**C1TC_HUMAN** Mass: 101558 Score: 39 Matches: 1(1) Sequences: 1(1) emPAI: 0.04

C-1-tetrahydrofolate synthase, cytoplasmic OS=Homo sapiens GN=MTHFD1 PE=1 SV=3

| Query | Score | Peptide |
|---|---|---|
| 660 | 39 | K.TPVPSDIDISR.S |

**H2AV_HUMAN** Mass: 13509 Score: 39 Matches: 1(1) Sequences: 1(1) emPAI: 0.34
Histone H2A.V OS=Homo sapiens GN=H2AFV PE=1 SV=3

| Query | Score | Peptide |
|---|---|---|
| 512 | 39 | R.GDEELDSLIK.A |

Proteins matching the same set of peptides:
H2AZ_HUMAN Mass: 13553 Score: 39 Matches: 1(1) Sequences: 1(1)
Histone H2A.Z OS=Homo sapiens GN=H2AFZ PE=1 SV=2

**FAS_HUMAN** Mass: 273423 Score: 39 Matches: 2(1) Sequences: 2(1) emPAI: 0.02
Fatty acid synthase OS=Homo sapiens GN=FASN PE=1 SV=3

| Query | Score | Peptide |
|---|---|---|
| 439 | 39 | K.AQVADVVVSR.W |
| 643 | 38 | R.SDEAVKPFGLK.V |

**RL22_HUMAN** Mass: 14787 Score: 38 Matches: 1(1) Sequences: 1(1) emPAI: 0.31
60S ribosomal protein L22 OS=Homo sapiens GN=RPL22 PE=1 SV=2

| Query | Score | Peptide |
|---|---|---|
| 672 | 38 | K.ITVTSEVPFSK.R |

**GBLP_HUMAN** Mass: 35076 Score: 38 Matches: 1(1) Sequences: 1(1) emPAI: 0.12
Guanine nucleotide-binding protein subunit beta-2-like 1 OS=Homo sapiens GN=GNB2L1 PE=1 SV=3

| Query | Score | Peptide |
|---|---|---|
| 1357 | 38 | K.IIVDELKQEVISTSSK.A |
